# Residual disease activity and treatment adjustments in psoriatic arthritis in current clinical practice

**DOI:** 10.1186/s13075-017-1424-8

**Published:** 2017-10-10

**Authors:** Leonieke J. J. van Mens, Marleen G. H. van de Sande, Inka A. Fluri, Sadaf Atiqi, Arno W. R. van Kuijk, Dominique L. P. Baeten

**Affiliations:** 1 Department of Clinical Immunology & Rheumatology, Amsterdam Rheumatology and immunology Center, Academic Medical Center/University of Amsterdam, Meibergdreef 9, 1105 AZ Amsterdam, The Netherlands; 2 Rheumatology, Amsterdam Rheumatology and immunology Center | Reade, Amsterdam, The Netherlands

**Keywords:** Psoriatic arthritis, Clinical practice, Treatment decisions, Residual disease activity, Treatment strategies

## Abstract

**Background:**

With expanding therapeutic possibilities for treatment of psoriatic arthritis (PsA) it will be increasingly important to determine residual disease and define when to adjust treatment. The rationale behind treatment decisions in current daily clinical practice and the relationship with residual disease activity has not been investigated. The aim of this study was to assess current clinical practice on defining residual disease and subsequent treatment decisions made in PsA patients.

**Methods:**

This cross-sectional study scored disease activity and treatment decisions prospectively in 142 consecutive PsA patients visiting the outpatient clinic for routine follow up. Disease activity parameters were scored by patient and the treating rheumatologist; the rheumatologist additionally registered his opinion on the presence of remaining disease activity despite current treatment (further mentioned as remaining disease) and subsequent treatment decisions.

**Results:**

Two thirds (90/142) of patients had remaining disease activity according to the treating rheumatologist. Almost half (46%) of these patients had moderate to high disease activity according to the clinical Disease Activity Index for Psoriatic Arthritis (cDAPSA). Residual disease activity was determined by joint disease and pain rather than by active psoriasis. Demographic and clinical features were similar between groups with or without residual disease. Among patients with remaining disease activity, 74% were treated with either a conventional synthetic disease-modifying anti-rheumatic drug (csDMARD) only or a first TNF-inhibiting biological agent, suggesting opportunities for treatment modification. However, treatment adjustment was initiated in only 21 (23%) of the 90 patients with residual disease. When comparing patients with remaining disease activity with and without treatment adjustment, we found no differences in objective disease activity measures, such as joint counts and patient scores. These data suggest that treatment is not adjusted in a large majority of patients with residual disease activity, although options for treatment changes are available.

**Conclusions:**

Remaining disease activity is present in almost two thirds of patients with PsA when scored by the treating rheumatologist, but triggers treatment adjustment in only a minority. Further research to understand why disease activity does not lead to treatment adjustment is required to enable implementation of treatment strategies in clinical practice.

## Background

Treatment options for psoriatic arthritis (PsA) increased significantly in the last decade with leflunomide and tumor necrosis factor (TNF) inhibitors (TNFi) becoming part of our standard therapeutic arsenal [1]. Several other new treatments became available in recent years, including ustekinumab, apremilast, and secukinumab [2–5]. Additionally, the first trial comparing target-steered treatment vs standard care in PsA, the Tight Control of Psoriatic Arthritis (TICOPA) trial, demonstrated that not only the availability of treatments but also the strategy on how to use these treatments determines the clinical outcome. The treat-to-target arm of the study shows significantly better outcomes in peripheral arthritis, skin, and patient reported outcomes in comparison with the standard-care arm, with more patients reaching a minimal disease activity state in the treat-to-target arm [6]. Therefore, the combination of additional treatment options and better treatment strategies holds great promise to improve the outcome in PsA in daily clinical practice.

Intriguingly, the few available publications on real-life clinical PsA practice indicate that not many patients are in a minimal disease state despite treatment [7]. Potential reasons for residual disease activity in this group of patients could be categorized as follows: (1) patients not responding to all available treatments - for this category, new treatment options obviously open up new perspectives; (2) patients in whom comorbidities, side effects, and/or incompliance result in the inability or unwillingness to use available medication - in this case, new treatments may not have an additional value; and (3) patients in whom the currently available treatments are not optimally used - this category could benefit from stricter definition and implementation of treatment strategies.

To quantify the presence of remaining disease activity in PsA in daily clinical practice and understand the underlying reasons, we set up a cross-sectional cohort documenting disease activity and treatment use in consecutive patients with PsA and analyzed the treatment decisions that were made by the treating rheumatologist. Importantly, the available treatment options in clinical practice during the inclusion period of this study were limited to conventional disease modifying anti-rheumatic drugs (csDMARDs), such as methotrexate and leflunomide, and TNFi. Ustekinumab became available for use in clinical practice just before this study and was therefore not a common drug to prescribe at the time.

## Methods

This observational cross-sectional study was conducted at the rheumatology outpatient clinics of two centers in Amsterdam (AMC and Reade) between October 2014 and September 2015. Sixteen participating rheumatologists recruited consecutive patients with PsA visiting their outpatient clinic, including a total of 152 patients. The inclusion criteria were (1) clinical diagnosis of psoriatic arthritis, (2) age 18 years or older, (3) disease duration of at least 6 months, (4) fulfillment of the Classification Criteria for Psoriatic Arthritis (CASPAR) [8], and (5) current or previous treatment with synthetic and/or biological DMARD therapy. Patients currently participating in a clinical trial were excluded from the study. This observational study did not require ethical approval and/or patient informed consent as the Medical Research Involving Human subjects Act (WMO) does not apply to these types of studies in the Netherlands, as confirmed by the Ethics Committee of the Academic Medical Center/University of Amsterdam. The study was conducted in accordance with the Declaration of Helsinki (2008) and International Conference on Harmonization (ICH)/Good Clinical Practice (GCP) standards.

### Data collection

Data on demographics, disease characteristics, current treatment, treatment history, and medical history were extracted from the patient files by the study physician (LvM). To reflect decision-making in real-life clinical practice, the disease activity scores and the statements on the presence of remaining disease and the decisions on treatment adjustment were made by the treating rheumatologist during a routine clinical visit. At the time of the visit the treating rheumatologist performed a physical examination, collected outcome measures, and answered questions on remaining disease activity and treatment modifications. Physical assessments included scoring of swelling and tenderness of the joints (swollen joint count (SJC)66/tender joint count (TJC)68), an enthesitis count, recording the number of enthesial sites with enthesitis according to the rheumatologist, and a dactylitis count. Disease activity measures were the Physician global Assessment on disease activity (VASPhysGlobal) and a Physician Assessment of psoriatic skin activity (VASPhysSkin) on a visual analog scale (VAS) of 0–10 cm and the Bath Ankylosing Spondylitis Activity Disease Index (BASDAI). The following questions were answered by the rheumatologist. Do you think there is remaining disease activity in this patient, despite current treatment regimen? (further mentioned in this manuscript as “remaining disease”). Will you start additional therapy or change current therapy for the remaining disease activity? If yes, what will you start or change? If no, what is the reason not to treat this remaining disease activity?

Patients completed the Patient Global Assessment of Disease Activity (VASPtGlobal) on a VAS of 0–10 cm and the Patient Assessment Of Pain (VASPtPain) on a VAS of 0–10 cm. To grade the severity of the disease activity in a composite index, we used the clinical Disease Activity in PsA score (cDAPSA) calculated as SJC66 + TJC68 + VASptglobal + VASptPain and divided by categories: remission, cDAPSA ≤4; low disease activity, cDAPSA >4 and ≤13; moderate disease activity, cDAPSA >13 and ≤27; and high disease activity, cDAPSA >27 [9, 10].

### Data analysis

Data are presented as mean and standard deviation (SD) or as median and interquartile range (IQR) where applicable. Statistical comparisons between remaining disease groups and treatment yes/no groups were performed using the *t* test or, alternatively, the Mann-Whitney *U* test when data were not normally distributed. Statistical tests were two-sided and *P* values <0.05 were considered statistically significant. Analysis was performed in SPSS (V22.0).

## Results

A total of 152 patients were included, and data scored by the rheumatologist and patient scores were available for 142 patients. The remaining 10 were excluded from analysis as either the physician or the patient data were missing. Disease characteristics and demographics are shown in Table 1.

**Table 1:** Clinical characteristics and disease activity of patients with and without residual disease activity

	Total group (*n* = 142)	Residual disease activity according to rheumatologist (*n* = 90)	No residual disease activity according to rheumatologist (*n* = 52)	Residual disease vs no residual disease *P* value
Age mean (SD)	53.0 (12.6)	53.6 (6.3)	51.9 (13.6)	0.000
Male/female *n*/*n*	92/60	53/37	33/19	0.865
Disease duration diagnosis mean (SD)	10.3 (7.9)	11.0 (9.2)	10.1 (6.8)	0.494
on treatment > 6 months, %	93	86	94	0.618
Current only DMARD user *n* (%)	110 (49)	52 (58)	23 (44)	0.032
Current first TNF user *n* (%)	32 (22)	14 (16)	18 (35)	
Current > 1 TNF user *n* (%)	35 (24)	24 (27)	11 (21)	
Swollen joints	0 (0–0)	0 (0–1)	0 (0–0)	0.000
SJC of 0, *n* (%)	111 (77)	63 (70)	49 (94)	
SJC of 1, *n* (%)	10 (7)	8 (9)	2 (4)	
SJC of 2, *n* (%)	5 (3)	5 (6)	0 (0)	
SJC of ≥ 3, *n* (%)	15 (10)	14 (16)	1 (2)	
Tender joints	1 (0–2)	1 (0–3)	0 (0–0)	0.000
TJC of 0, *n* (%)	82 (57)	36 (40)	46 (88)	
TJC of 1, *n* (%)	21 (13)	16 (18)	5 (10)	
TJC of 2, *n* (%)	12 (13)	12 (13)	0 (-)	
TJC of 3, *n* (%)	25 (17)	24 (27)	1 (2)	
Number of dactylitic digits	0 (0–0)	0 (0–0)	0 (0–0)	0.017
Dactylitis count of 1, *n* (%)	8 (6)	8 (9)	0 (-)	
Number of enthesitis points	0 (0–0)	0 (0–0)	0 (0–0)	0.001
Enthesitis count of 1, *n* (%)	14 (10)	14 (16)	0 (-)	
Enthesitis count of 2–4, *n* (%)	2 (1)	2 (2)	0 (-)	
VAS physician skin severity	1 (0–2)	1 (1–2.5)	1 (0–1)	0.000
VAS physician overall disease activity	1 (1–3)	2 (1–4)	1 (0–1)	0.000
VAS patient global disease activity	2 (1–6)	5 (2–7)	1 (0–2)	0.000
VAS pt pain	2 (1–6)	5 (2–7)	1 (0–2)	0.000
BASDAI	2,65 (1–4.8)	4.1 (2.5–5.9)	0.9 (0.4–1.8)	0.000
cDAPSA remission, *n* (%)	45 (32)	9 (10)	36 (69)	0.000
cDAPSA low disease activity, *n* (%)	49 (35)	34 (38)	15 (29)	
cDAPSA moderate disease activity, *n* (%)	35 (25)	35 (39)	0 (-)	
cDAPSA high disease activity, *n* (%)	6 (4)	6 (7)	0 (-)	
cDAPSA missing, *n* (%)	7 (5)	6 (7)	1 (2)	

Values are median (IQR) unless stated otherwise. Significance of the comparisons was determined by independent sample *t* test for continuous variables and the Mann-Whitney *U* test for non-normally distributed variables. The comparisons within cDAPSA groups were determined by the Kruskall-Wallis test

*csDMARD* conventional synthetic disease-modifying anti-rheumatic drugs, *BASDAI* Bath Ankylosing Spondylitis Disease Activity Index, *TNFi* tumor necrosis factor inhibitor, *SJC* swollen joint count, *TJC* tender joint count, *VAS* visual analog scale, *cDAPSA* clinical Disease Activity in PsA score

We first analyzed the disease activity present in patients with or without residual disease according to their treating rheumatologist. Fifty-two patients were considered by the treating rheumatologist not to have remaining disease activity (Table 1). These patients indeed had low disease activity in all measured objective disease domains: there were only three patients with a swollen joint count >0, three patients with a tender joint count >0, and no patients at all with dactylitis or enthesitis. The median skin severity score was 1 on the 10-point scale. This was confirmed by patient-reported severity and pain scores (median VASpatient global and VASpt pain score of 1 out of 10). Accordingly, all patients were in remission (36/52) or low disease activity (15/52) (there was insufficient data on one patient to calculate the cDAPSA) as defined by the cDAPSA composite score.

Of the 142 patients, 90 (63%) were considered in the opinion of the treating rheumatologist to have remaining disease activity (Table 1). This was supported by disease activity across all measured domains, with 31% of patients having at least one swollen joint (including 16% with three or more swollen joints), 60% having one or more tender joints, 18% having enthesitis, 9% having dactylitis, and 30% who had a VASphys skin score >2. This was reflected by higher scores in patient-reported severity of pain and in the global health score (median score of 5/10). The cDAPSA composite score indicated that 46% of these patients had moderate to high disease activity. Overall, these data indicate that almost two thirds of the patients had some kind of objective residual disease activity, with almost half of those having even moderate to high disease activity. The remaining disease activity was mainly determined by joint disease and pain rather than by skin psoriasis, partly driven by an overall low burden of skin psoriasis in this cohort.

In order to better define which patients had remaining disease activity, we compared demographic and clinical features between those with and without residual disease activity. There were no differences in gender, disease duration, comorbidity, current treatment duration, or number of previously used csDMARDS. Residual disease activity was more frequently reported in patients treated with a csDMARD only (66%) or a second TNFi (69%) in comparison with patients on their first TNFi (44%) (*P* = 0.019). As 74% of the patients with residual disease activity were currently treated with either a csDMARD only or a first TNFi, this suggests that treatment modification could be an option.

We asked the rheumatologist whether the presence of remaining disease activity would lead to a treatment adjustment. Of the 90 patients with remaining disease activity, treatment was modified in only 21 (23%). These treatment adjustments included start of analgesic treatment (*n* = 6), local or intramuscular corticosteroid therapy (*n* = 5), switch of csDMARD to another csDMARD (*n* = 4), referral to other specialists (hand surgeon, orthopedic surgery) (*n* = 2), start of a second and a third TNFi in a patient already using TNFi treatment (*n* = 2), addition of a csDMARD to current TNFi therapy (*n* = 1), and start of a TNFi in a patient only using csDMARDs (*n* = 1).

In order to understand why treatment adjustment was only initiated in 23% of the patients with remaining disease activity, we compared those with and without treatment adjustment for clinical and demographic features (Table 2). With the exception of VAS physician (median 3 (IQR 2–5) vs 2 (2–3), *P* = 0.007), none of the disease activity measures were different between the two groups, not even objective measures such as the SJC. Also demographic features such as gender, disease duration, and treatment durations were similar (Table 2). However, treatment was less frequently changed in patients already treated with a second TNFi (in 3/24 (13%) of patients) in comparison with the csDMARD only or first TNFi groups (15/51 (28%) and 4/14 (29%), respectively).

**Table 2:** Disease activity in patients with residual disease activity resulting or not resulting in additional treatment

	Total group (*n* = 90)	Additional treatment (*n* = 21)	No additional treatment (*n* = 69)	Additional treatment vs No additional treatment *P* value
Age, mean (SD)	53.6 (12.2)	52.6 (10.8)	53.9 (12.7)	0.270
Male/female, *n*/*n*	53/37	15/6	38/31	0.167
Disease duration diagnosis mean, (SD)	11.0 (9.2)	10.8 (8.6)	11.2 (9.4)	0.837
On treatment > 6 months, %	92	90	93	0.057
Swollen joints	0 (-0–1)	0 (0–1.5)	0 (0–1)	0.359
SJC of 0, *n* (%)	63 (70)	13 (62)	50 (72)	
SJC of 1, *n* (%)	8 (9)	3 (14)	5 (7)	
SJC of 2, *n* (%)	5 (6)	2 (10)	3 (4)	
SJC of ≥3, *n* (%)	14 (16)	3 (14)	11 (16)	
Tender joints	1 (0–3)	1 (0–2)	1 (0–3)	0.923
TJC of 0, *n* (%)	36 (40)	7 (33)	29 (42)	
TJC of 1, *n* (%)	16 (18)	8 (38)	8 (12)	
TJC of 2, *n* (%)	12 (13)	2 (10)	10 (15)	
TJC of ≥3, *n (%)*	25 (27)	4 (20)	21 (30)	
Number of dactylitic digits	0 (0–0)	0 (0–0)	0 (0–0)	0.310
Dactylitic digits ≥1, *n* (%)	8 (9)	3 (14)	5 (8)	
Number of enthesitis points	0 (0–0)	0 (0–0)	0 (0–0)	0.384
Enthesitis points ≥1, *n* (%)	16 (18)	5 (24)	11 (16)	
VAS physician skin severity	1 (1–3)	2 (1–3.5)	1 (1–2)	0.478
VAS physician overall disease activity	2 (1–4)	3 (2–5)	2 (1–3)	0.007
VAS patient global disease activity	5 (2–7)	6 (3–7)	5 (2–7)	0.157
VAS pt pain	5 (2–7)	6 (3–7)	4 (2–7)	0.225
BASDAI	4.1 (2.5–5.9)	4.2 (2.7–5.8)	3.7 (2.5–5.9)	0.907
cDAPSA remission, *n* (%)	9 (10)	1 (5)	8 (12)	0.594
cDAPSA low disease activity, *n* (%)	34 (38)	7 (33)	27 (39)	
cDAPSA moderate disease activity, *n* (%)	35 (39)	11 (52)	23 (33)	
cDAPSA high disease activity, *n* (%)	6 (7)	2 (10)	4 (6)	
cDAPSA missing, *n* (%)	6 (7)	0	6 (9)	

Numbers are median (IQR) unless stated otherwise. Significance of the comparisons is determined by independent sample *t* test for continues variables and the Mann-Whitney *U* test for non-normally distributed variables. The comparisons within the cDAPSA groups were determined by the Kruskall-Wallis test

*SJC* swollen joint count, *TJC* tender joint count, *VAS* visual analog scale, *BASDAI* Bath Ankylosing Spondylitis Disease Activity Index, *cDAPSA* clinical Disease Activity in PsA score

We additionally investigated the reasons not to adjust treatment. In 39/69 (57%) patients, the rheumatologist judged the complaints as “minor”. This group included 8 patients with a SJC ≥1, 19 with a TJC ≥1, and 6 with an enthesitis. cDAPSA scores categorized these patients as having moderate disease activity (*n* = 12), low disease activity (*n* = 19), and remission (*n* = 5) (with 4 patients having incomplete data to calculate the cDAPSA score). In the remaining 30 patients the following reasons were reported: patients’ preference not to adjust medication (10/69 (14%)); absence of additional treatment options (5/69 (7%)) (these patients were all currently treated with a second or third TNFi); lack of compliance and/or adverse events(5/69 (7%)); and other reasons (20/69 (29%)) (more than one reason could be reported per patient). Other reasons included: disease activity reflects symptoms of comorbidity instead of the PsA (non-inflammatory joint problems/disease including osteoarthritis of the knee/hand, trigger finger, meniscal tear, and recent fracture) (*n* = 4); awaiting therapeutic effect of recent change (*n* = 3); side effects resulted in a disruption of treatment; recently restarted therapy (*n* = 3); no therapeutic options left for this patient (*n* = 2); first awaiting results of magnetic resonance imaging (MRI) (*n* = 1); task of the dermatologist (*n* = 2); comorbidity prohibits treatment intensification (*n* = 2; one patient was recently diagnosed with breast cancer and one patient had undergone surgery complicated by insufficient wound healing); side effects limit use of optimal dosage for treatment (*n* = 1); patient is incompliant to therapy (*n* = 1). Overall, judgment by the rheumatologist and/or patient rather than objective hurdles to intensify treatment (absence of additional treatment options, lack of compliance, intolerance) drove the decision not to modify treatment despite the presence of residual disease activity.

## Discussion

This prospective, cross-sectional study in 142 patients with PsA in daily clinical practice showed that despite being on stable treatment and follow up, almost two third of the patients were considered by the treating rheumatologist to have residual disease activity. Almost half (46%) of these patients had moderate to high disease activity according to the cDAPSA. Residual disease activity was determined by joint disease and pain rather than by active psoriasis. Our findings are perfectly aligned with recently published data from Michelsen et al., showing that only a few patients with PsA fulfilled remission criteria and only 25% fulfilled minimal disease activity (MDA) in daily clinical practice [7]. Whereas these data suggest that a majority of patients may benefit from treatment modification and/or intensification, certainly in an era with emerging novel treatment options, treatment was adjusted in only 21 patients with PsA (23%) with residual disease activity in our cohort. While the cDAPSA showed moderate to high disease activity in almost half of these patients, the rheumatologists reported in a majority of patients that (a) the residual disease was not substantial enough to justify treatment adjustment or (b) that the patient did not wish to adjust treatment. When comparing patients with remaining disease activity with and without treatment adjustment, we found no differences in objective disease activity measures, such as joint counts and patient scores. And albeit remaining disease was more prevalently found in patients treated with either csDMARDs alone or a second TNFi treatment, these groups were not more likely to receive a treatment adjustment. These data suggest that treatment is not adjusted in a large majority of patients with residual disease activity, although options for treatment changes are still available.

The only published treat-to-target study in PsA (TICOPA) reported that despite the clear protocol stating that treatment be adjusted when MDA was not fulfilled, in 37% no treatment adjustment followed [6]. Reported reasons for non-escalation (and not following the TICOPA study protocol) were (1) recent start of current therapy, (2) comorbidity, (3) no therapeutic options left, and (4) unable to tolerate the escalated dose. In our cohort, only a few patients had started or adjusted treatment recently or had severe comorbidity resulting in contraindications for TNFi or csDMARDs. As in TICOPA, the absence of therapeutic options was a decisive factor in a fraction of patients, as we observed more patients with residual disease activity in the group treated with a second TNFi, keeping in mind the new treatments such as ustekinumab only recently came to market and apremilast and secukinumab were not available at the time of our study. Intriguingly, however, residual disease activity was more frequently reported not only in patients treated with a second TNFi (69%), or first TNFi (44%), but also in patients on a csDMARD only (66%). In the latter group, the addition of a TNFi was at that time a very obvious, well-validated and recommended therapeutic alternative, which was not used despite ongoing disease activity [5, 11]. Important to note, in the Netherlands csDMARDS, TNFi, and the newer treatments are approved treatments. They are available and reimbursed to all patients with active PsA when prescribed by a rheumatologist, therefore payer issues can be excluded as an argument for not adjusting treatment.

Collectively, these data suggest that absence of treatment modification/intensification was not due to the absence of therapeutic options in a vast majority of patients. Alternative reasons for not adjusting treatment in patients with PsA with residual disease activity despite additional therapeutic options could include (1) lack of structural disease activity assessment in clinical practice, (2) limited availability of evidence that aggressive treatments result in improved short-term and long-term clinical outcomes, and (3) poor implementation of guidelines and treatment strategies in clinical practice.

As to lack of structural disease activity assessment in clinical practice, Coates et al. reported that PsA-specific measurements are not often used in routine clinical practice and that a target for treatment is defined in less than half of the visits [12]. Measuring activity in all domains and consensus on the definition of residual disease activity are warranted here as guidelines recommend “to achieve the lowest possible level of disease activity in all domains of disease”. Although the present study required a systematic evaluation of the different domains of PsA (arthritis, pain, enthesitis, dactylitis, skin) the rheumatologist was not asked to integrate these data in a comprehensive disease activity measure. Relying exclusively on the activity of the joints and/or more global questions, could potentially explain why the rheumatologists considered the complaints as minor in 57% of the patients with PsA with residual disease activity. It would be interesting to study if the use of a comprehensive measure such as MDA or a treat-to-target strategy based on MDA or DAPSA/cDAPSA would change the rheumatologist’s opinion on disease activity in the same patients.

A second factor to consider is the familiarity of the rheumatologist with the evidence that profound disease suppression is associated with better short-term and long-term outcomes in PsA. MDA is a well-validated measure [13]. Post-hoc analyses show that a sustained MDA state upon treatment results in better clinical and radiological outcomes [14, 15]. The TICOPA trial confirmed the benefit of the treat-to-target approach in a randomized clinical trial with better clinical and patient-reported outcomes in the treat-to-target arm compared with standard clinical care [6]. Importantly, the data from TICOPA became available after the inclusion of patients in the present study was completed. It would be interesting to assess if the availability of these new data would change the opinion of the rheumatologist.

The third and final factor is to what extent guidelines and treatment strategies are really implemented in daily clinical practice. The implementation of guidelines in rheumatology clinical practice has been shown to be a challenge. Studies in rheumatoid arthritis indicate that there is a discrepancy between the reported acceptance of guidelines and the application of these in clinical practice [16, 17]. The IRIS study even showed that despite participation in 2 years of educational training, rheumatologists seem to be reluctant to apply recommendations in real-life clinical practice [18]. These factors could play a role in PsA as well, and attention to the implementation of concepts and guidelines may even be the most important factor to consider when developing treatment strategies for PsA.

As this was a real-life, non-interventional study, the limitations to our study include (a) we did not have any influence on the treatment strategy applied by the rheumatologists; however, csDMARDS (oral and subcutaneous) and TNFi are available and reimbursed to all patients with active PsA when prescribed by a rheumatologist; (b) we incorporated measurements feasible for use in our current clinical practice and therefore did not include a measurement of nail disease or physical functioning, and did not collect laboratory or radiological data; (c) not including multiple additional measurements resulted in an inability to calculate more comprehensive measures of PsA disease activity such as the Psoriatic Arthritis Disease Activity Score (PASDAS) or MDA, capturing more domains of the disease. Nevertheless, the DAPSA and cDAPSA are measurements suggested for use in the treat-to-target setting of PsA [10]; and (d) we did not have sufficient power to study different subsets of patients.

## Conclusions

Within the limits of this cross-sectional evaluation, we conclude that residual disease activity was present in almost two thirds of patients with PsA in a daily clinical practice cohort but triggered treatment adjustment in only a quarter of those patients. Subjective opinions of the rheumatologist and/or the patient rather than comorbidities or a lack of treatment options drove the decision not to adjust treatment despite residual disease activity. With the increasing availability of novel drugs to treat PsA and the ongoing efforts on a consensus for treatment target, better understanding as to why residual disease activity does not lead to treatment adjustment in clinical practice is a third important pillar in developing and implementing treatment strategies to improve outcome in PsA.
